# A novel approach of utilization of the fungal conidia biomass to remove heavy metals from the aqueous solution through immobilization

**DOI:** 10.1038/srep36546

**Published:** 2016-11-16

**Authors:** Chun-Xiang Cai, Jian Xu, Nian-Fang Deng, Xue-Wei Dong, Hao Tang, Yu Liang, Xian-Wei Fan, You-Zhi Li

**Affiliations:** 1 State Key Laboratory for Conservation and Utilization of Subtropical Agro-bioresources; Key Laboratory of Ministry of Education for Microbial and Plant Genetic Engineering; College of Life Science and Technology, Guangxi University, 100 Daxue Road, Nanning, Guangxi 530004, P. R. China; 2 Hezhou University, 18 Xihuan Road, Hezhou, Guangxi 54289, P. C. China

## Abstract

The biomass of filamentous fungi is an important cost-effective biomass for heavy metal biosorption. However, use of free fungal cells can cause difficulties in the separation of biomass from the effluent. In this study, we immobilized the living conidia of the heavy metal-resistant *Penicillium janthinillum* strain GXCR by polyvinyl alcohol (PVA)-sodium alginate (SA) beads to remove heavy metals from an aqueous solution containing a low concentration (70 mg/L) of Cu, Pb, and Cd. The PVA-SA-conidia beads showed perfect characters of appropriate mechanical strength suitable for metal removal from the dynamic wastewater environment, an ideal settleability, easy separation from the solution, and a high metal biosorption and removal rate even after four cycles of successive sorption-desorption of the beads, overcoming disadvantages when fungal biomasses alone are used for heavy metal removal from wastewater. We also discuss the major biosorption-affecting factors, biosorption models, and biosorption mechanisms.

Heavy metals in aqueous solutions often originate from effluents discharged from many industries, and these heavy metals are usually characterized by their hazardous effects, persistency, and tendency to accumulate^1^. One of consequences of improper and/or untreated discharge of such wastewater is contamination of surface- and ground-water resources^2^. Therefore, removal of heavy metals from the wastewater has become important for human and environmental health. However, conventional treatment technologies, such as precipitation and coagulation, of wastewater with low concentrations of heavy metals are usually limited because of cost constraints^3^. In addition, with growing environmental awareness, demand for eco-friendly and cost-effective biosorbent-based treatment technology is increasing^2^,^4^.

Microbial biomass-based metal biosorption techniques, especially those employing filamentous fungi, are of low cost in comparison to sorption on commercial ion-exchange resins, activated carbon, and metal oxides^3^. Fungal biosorption also offers effective technology for metal recovery from aqueous solutions^4^, with the biomass of a great array of filamentous fungi^4^,^5^,^6^,^7^,^8^,^9^,^10^,^11^,^12^,^13^,^14^,^15^,^16^,^17^,^18^. Typically, two types of filamentous fungi biomass are being adopted in heavy metal removal in studies, living or inactivated biomass^4^,^13^,^14^. However, metal biosorption by dead microbial biomass is only surface-area limited passive adsorption^19^, whereas the application of living cells is obviously advantageous via diverse internal metabolism-dependent metal-resistance mechanisms such as metal detoxification and bioaccumulation^13^,^20^ with sustained cell growth although the costs associated with maintaining living cells reduce cost-effectiveness^4^. These biologically-mediated processes are often termed ‘biosorption’ rather than bio-adsorption or bio-uptake^21^. However, the living cells used are likely subjected to both toxicity form heavy metals and adverse operating conditions^3^. In this case, growing metal-resistant cells would be preferable in metal removal^13^. The conidia of the filamentous fungi are in close proximity to bacterial cells in shape and size, but they have a unique advantage over bacteria because an individual conidium can produce much higher amounts of mycelial biomass than single bacterial cell. However, the small particle size, elevated dispersibility, and high buoyancy of fungal cells make it difficult to separate and recover their biomass from the effluent in industrial applications^3^. One of the best choices to solve these problems is to immobilize or pelletize biomass^3^. In our experience, directly immobilizing large amounts of mycelial biomass onto support materials is not the best choice because it needs special pulverization. However, immobilizing the conidia produced by the fungal mycelia is substantially more preferable because the conidia have a grain-like morphology that is easily embedded and subsequently grow a lot of mycelial biomass under certain conditions. However, the application of the fungal conidia immobilized within polymer beads to heavy metal removal should take into consideration of physicochemical conditions, optimization of the parameters of the biosorption process, recovery and reuse of immobilized cells^4^, depending on adsorption systems. To our knowledge, the mechanisms of heavy metal biosorption by immobilization of the fungal conidia are largely unknown.

Previously, we reported a strain of filamentous fungus, *Penicillium janthinillum* strain GXCR, which has very high resistance to multiple heavy metals and strong metal biosorption by the mycelial biomass^22^. In this study, we investigate heavy metal removal by using GXCR conidia immobilized in polyvinyl alcohol (PVA) and sodium alginate (SA) to develop a new technology to remove the heavy metals from wastewater, while also characterizing the mechanisms associated with heavy metal removal.

## Results

### The optimum conditions of preparation of beads for embedding conidia

Before heavy metal biosorption tests using the beads immobilizing GXCR conidia, it is necessary to optimize physical properties such as, strength, rigidity, and porosity of the beads^23^. By orthogonal experiments (Table 1), the optimal conditions for preparation of the beads in this study were determined to be 2% PVA, 3% SA, 1% H_3_BO_3_, and 3% CaCl_2_ through cross-linking for 20 min (Table 1). Under these conditions, the beads easily formed, and showed a better settleability and didn’t stick together each other. If as loading weight, the average mechanical strengths per a bead were estimated to be 31 g for PVA-SA-conidia beads and 21 g for PVA-SA beads, respectively.

### Changes in metal biosorption capacity and removal efficiency with biosorption duration

**Figure 1:**
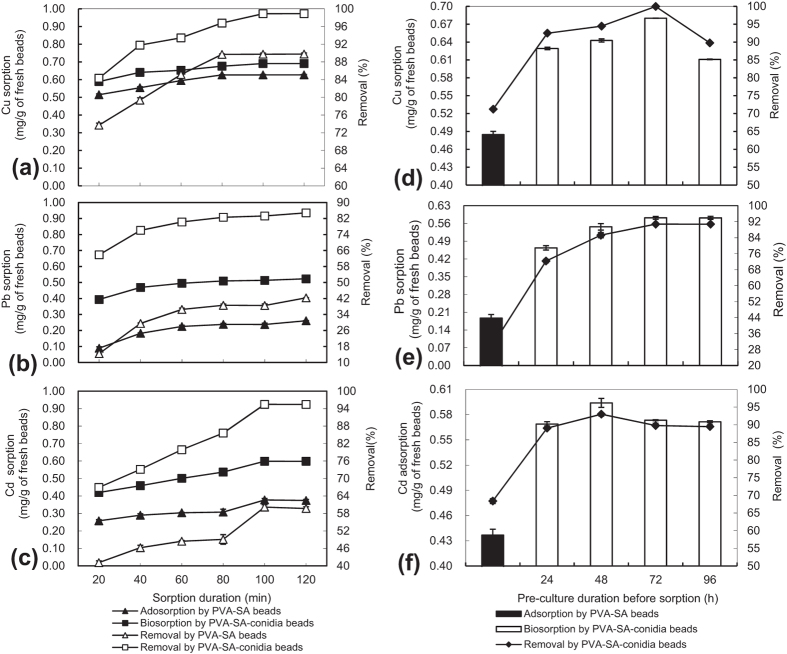
Changes in sorption and removal of Cu (**a**), Pb (**b**), and Cd (**c**) by the beads over sorption duration; and sorption and removal of Cu (**d**), Pb (**e**), and Cd (**f**) by the beads over the pre-culture duration. For assays to detect changes in sorption and removal of the metals with sorption duration, the beads with a diameter of 3.2 mm were pre-cultured for 48 h by shaking at 200 rpm in LPD and then thoroughly rinsed with sterilized water before biosorption. The sorption was conducted in a single metal (70 mg/L) solution of unadjusted natural pH and containing 5 g beads by shaking at 200 rpm at 32 °C. Following the same procedures, sorption and removal of the metals over pre-culture duration of the beads were assayed. Each data point represented the average mean ± standard deviation (SD) from three independent experiments performed in parallel. LPD, liquid potato dextrose. PVA, polyvinyl alcohol. SA, sodium alginate. SD, standard deviation.

As shown in Fig. 1a–c, the metal biosorption amount and metal removal rate of Cu, Pb, and Cd by the PVA-SA-conidia beads increased with biosorption time. After a 100 min sorption treatment by PVA-SA-conidia beads, Cu biosorption and removal efficiency were up to 0.69 mg/g of beads and 98.9% (Fig. 1a), respectively; Pb biosorption and removal efficiency were up to 0.60 mg/g and 95.5% (Fig. 1b), respectively; and Cd biosorption and removal efficiency were up to 0.52 mg/g and 84.8% (Fig. 1c), respectively. Note that the control PVA-SA beads without conidia also showed metal adsorption, but the metal removal efficiency was significantly (*p* < 0.05) lower than that by the PVA-SA-conidia beads at each time point of the corresponding treatments (Fig. 1a–c).

### Changes in metal sorption capacity and removal efficiency with the pre-culture time of the PVA-SA-conidia beads

The growth status of embedded conidia in the beads likely affects subsequent metal biosorption capacity. Therefore, we investigated the effects of incubation time of PVA-SA-conidia beads on the biosorption before use. As a result, the PVA-SA-conidia beads showed higher Cu (Fig. 1d) and Pb biosorption after a 72-h pre-culture (Fig. 1e) and seemed to prefer Cd adsorption after a 48-h pre-culture (Fig. 1f).

### Changes in metal sorption capacity and removal efficiency with initial metal concentration in the solution

**Figure 2:**
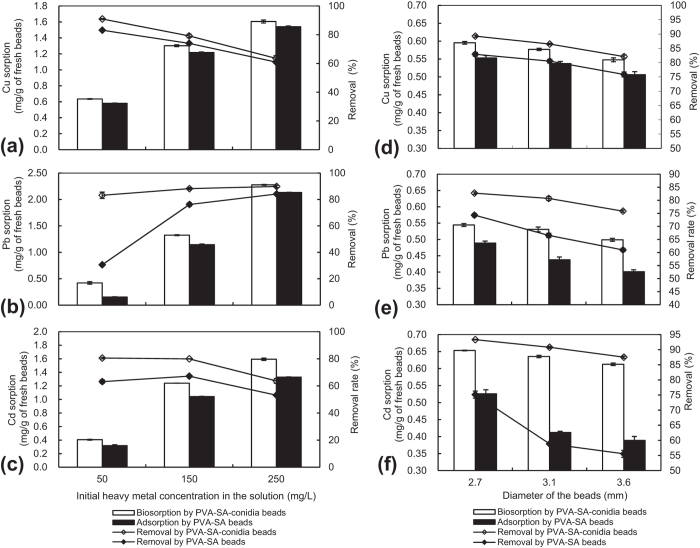
Sorption and removal of Cu **(a),** Pb **(b)**, and Cd **(c)** by the beads under different initial metal concentrations, and effects of bead size on sorption and removal of Cu **(d),** Pb **(e),** and Cd **(f)**. To determine the sorption and removal of the metals under different initial metal concentration, 3.2 mm diameter beads were pre-cultured for 48 h by shaking at 200 rpm in LPD and thoroughly rinsed in sterilized water before sorption. The sorption was conducted in a single metal (70 mg/L) solution of unadjusted natural pH and containing 5 g beads by shaking for 3 h at 200 rpm at 32 °C. To assay the effects of bead size on sorption and removal of the metals, the beads were pre-cultured for 48 h by shaking at 200 rpm in LPD and then fully rinsed by sterilized water before sorption. The sorption was conducted following the above-indicated procedures with conditions except the bead size. Each datum in the Figure was the average mean ± SD from three parallel independent experiments. LPD, liquid potato dextrose. PVA, polyvinyl alcohol. SA, sodium alginate. SD, standard deviation.

With increasing initial concentration of corresponding metals in the single metal-containing solution, the biosorption and adosorption for Cu, Pb, and Cd, respectively, by PVA-SA-conidia beads and by control PVA-SA beads increased (Fig. 2a–c). However, the removal rates of Cu (Fig. 2a) and Cd (Fig. 2c) gradually decreased by both types of the beads. Note that the removal rate of Pb was significantly (*p* < 0.05) increased by PVA-SA beads rather than PVA-SA-conidia beads (Fig. 2b).

### Changes in metal sorption capacity and removal efficiency with bead size

Interestingly, the sorption amounts and removal rates of Cu (Fig. 2d), Pb (Fig. 2e), and Cd (Fig. 2f) by either PVA-SA-conidia or control PVA-SA beads tended to gradually decrease with the increase in bead size if the total weight of used beads and the amount of embedded conidia for each bead remained unchanged. The significant difference (*p* < 0.05) occurred between the treatments of the beads at 2.7 and 3.6 mm in diameter (Fig. 2d–f).

### Changes in metal biosorption capacity and removal efficiency with initial pH in the solution

**Figure 3:**
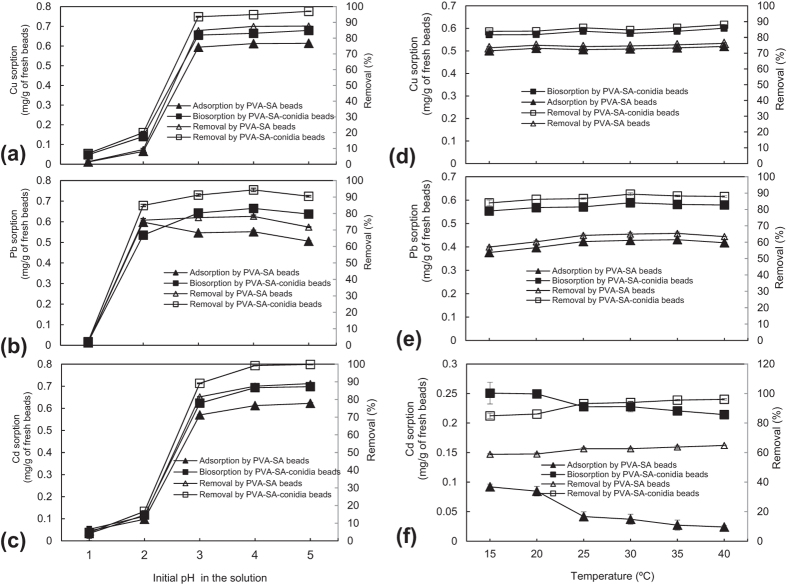
Changes in sorption and removal of Cu (**a**), Pb (**b**), and Cd (**c**) with varying initial solution pH, as well as changes in sorption and removal of Cu (**d**), Pb (**e**), and Cd (**f**) as a function of temperature. To determine sorption and the removal of the metals with initial pH in the solution, the beads were pre-cultured for 48 h by shaking at 200 rpm in LPD and then thoroughly rinsed by sterilized water before sorption. The sorption was conducted in a single metal (70 mg/L) solution of different initial pH values and containing 5 g beads of a 3.2 mm diameter by shaking incubation for 3 h at 200 rpm at 32 °C. To determine sorption and removal of the metals as a function of temperature, the beads were pre-cultured for 48 h by shaking at 200 rpm in LPD and then fully rinsed by sterilized water before sorption. The sorption was conducted following the above-indicated procedures under conditions except temperature changes. Each data point in the Figure represented the average mean ± SD from triplicate independent experiments. LPD, liquid potato dextrose. PVA, polyvinyl alcohol. SA, sodium alginate. SD, standard deviation.

With increasing initial pH from 1 to 5 in the solution, the sorption amounts and removal rates of Cu, Pb, and Cd by either PVA-SA-conidia or control PVA-SA beads significantly (*p* < 0.05) increased (Fig. 3a–c). The removal rates by PVA-SA-conidia beads at pH 5 were up to 97.0% for Cu (Fig. 3a), 90.5% for Pb (Fig. 3b), and 99.8% for Cd (Fig. 3c).

### Changes in metal sorption capacity and removal efficiency with temperature

With increasing temperature from 15 to 40 °C, the sorption amounts and removal rates of Cu (Fig. 3d) and Pb (Fig. 3e) by either PVA-SA-conidia or control PVA-SA beads were hardly affected. By using PVA-SA-conidia beads, the Cd biosorption started significantly (*p* < 0.05) decreasing at 25 °C, in the meantime, its removal rate significantly (*p* < 0.05) increased (Fig. 3f). However, with control PVA-SA beads, the Cd adsorption began to significantly (*p* < 0.05) decrease at 25 °C while the removal rate almost kept constant during increasing temperature (Fig. 3f).

### Changes in metal removal efficiency with times of bead reuse

**Figure 4:**
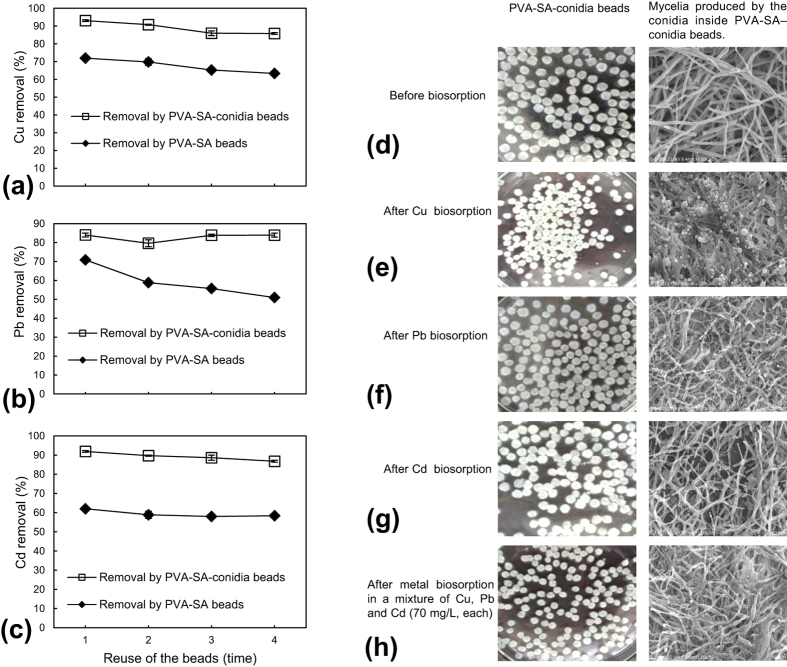
Changes in removal rate of Cu (**a**), Pb (**b**), and Cd (**c**) with times of reuse of the beads, and images of the PVA-SA-conidia beads, and mycelia inside the beads (**d–h**). To determine the changes in removal rate of the metals as a function of times of bead reuse, the beads were pre-cultured for 48 h by shaking at 200 rpm in LPD and then fully rinsed with sterilized water before sorption. The sorption was conducted in a single metal (70 mg/L) solution of natural pH and containing 5 g beads by shaking for 3 h at 200 rpm at 32 °C. After sorption, the the beads were regenerated for reuse following the procedures in Methods. Each data point in the Figure was the average mean ± SD from three independent experiments. To observe the beads and mycelia inside the beads, the beads were pre-cultured for 72 h by shaking at 200 rpm in LPD and then fully rinsed by sterilized water before sorption. The sorption was conducted in a single metal (70 mg/L) and multi-component metal (70 mg/L for each metal) solutions of unadjusted natural pH and containing 5 g beads of a 3.2 mm diameter by shaking incubation for 3 h at 200 rpm at 32 °C, respectively. The beads were photographed with an ordinary optical microscope. The mycelia inside the beads were observed by SEM. LPD, liquid potato dextrose. PVA, polyvinyl alcohol. SA, sodium alginate. SEM, scanning electron microscope.

Reuse of the metal adsorbents can reduce the cost of metal removal in practice. Therefore, we assayed biosorption after reuse of the PVA-SA-conidia beads in the single metal solution system containing Cu, Pb, and Cd. Consequently, the removal rate by the conidia beads was 85.8% for Cu (Fig. 4a), 84.0% for Pb (Fig. 4b), and 86.8% for Cd (Fig. 4c) after four successive cycles of sortpion and desorption.

### Mycelial patterns inside the beads before and after metal biosorption

The conidia in PVA-SA-conidia beads could germinate and produce well-developed mycelia inside the beads before biosorption (Fig. 4d). When conducting biosorption tests in the single metal solution, there were spherical components that attached on the mycelial surface or disturbed in the mycelia-formed porosity space after Cu biosorption (Fig. 4e), but some small irregularly shaped deposits adhered to the mycelial surface after Pb biosorption (Fig. 4f). No unusual substance was found on the mycelial surface or in the mycelia-formed space after Cd biosorption (Fig. 4g). After the biosorption in the multi-component metal solution of Cu, Pb, and Cd, the mycelial case in the beads (Fig. 4h) was similar to that in the beads after Cd biosorption (Fig. 4g). After sorption, the surfaces of the mycelia inside of the PVA-SA-conidia beads (Fig. 4e–h) appeared very coarse when compared smooth surfaces of the mycelia inside the beads before sorption/desorption treatments (Fig. 4d).

### Functional molecular groups for biosorption of metals

**Figure 5:**
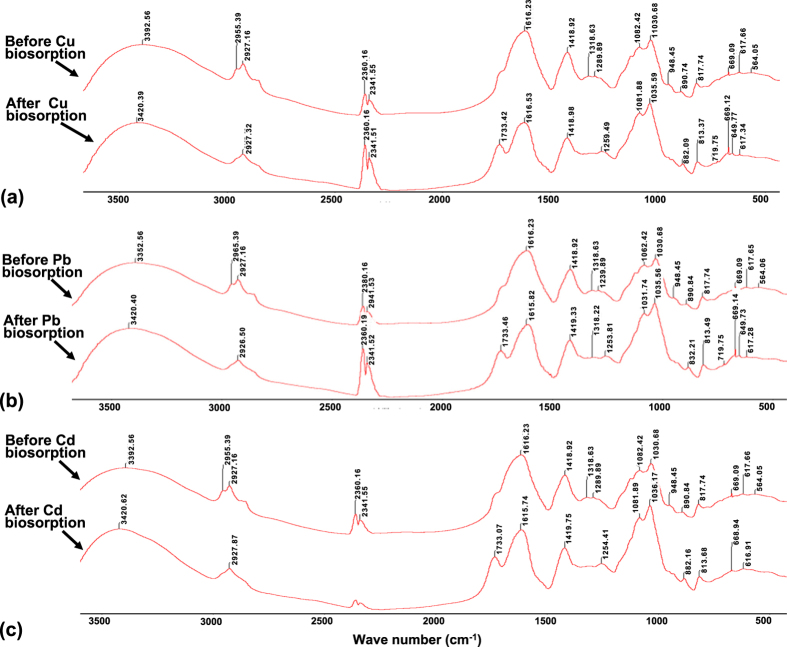
The IS values before and after biosorption of Cu (**a**), Pb (**b**) and Cd (**c**) by the PVA-SA-conidia beads. To detect the IS values, the beads were pre-cultured for 48 h by shaking at 200 rpm in LPD and then fully rinsed by sterilized water before sorption. The biosorption was conducted a single metal (70 mg/L) solutions of unadjusted natural pH and containing 5 g beads of a 3.2 mm diameter by shaking incubation for 3 h at 200 rpm at 32 °C. The IS was recorded by the FT-IR spectrophotometer. FT-IR, Fourier transform infrared. IS, infrared spectra. LPD, liquid potato dextrose. PVA, polyvinyl alcohol. SA, sodium alginate.

To identify functional groups for metal biosorption, we conducted the infrared spectra (IS) analysis. Consequently, based on changes in positions of infrared peaks of PVA-SA-conidia beads before and after biosorption of Cu (Fig. 5a), Pb (Fig. 5b), and Cd (Fig. 5c), the obviously functional groups for biosorption of Cu and Pb were: -OH, C = O, CH_3_, CH_2_, PO_3,_ and 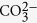. In addition, a unique 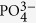-PO_4_ was also found to be associated with Cd biosorption (Table 2).

### Determination of the sorption isotherm

**Figure 6:**
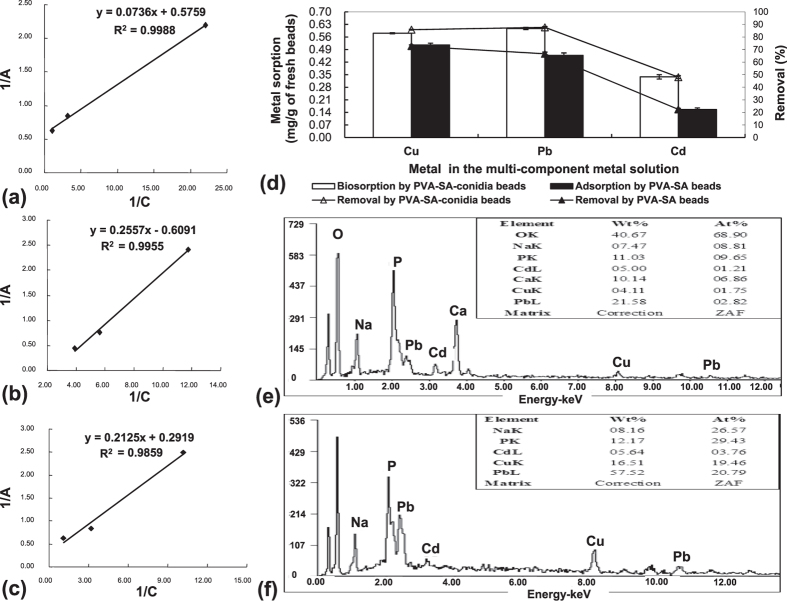
The Langmuir isotherm fitting results for biosorption of Cu (**a**), Pb (**b**) and Cd (**c**) by the PVA-SA-conidia beads, respectively; metal sorption and removal from the multi-component metal solution (**d**); and energy spectra of control PVA-SA beads (**e**), and PVA-SA-conidia beads after biosorption (**f**), respectively. The Langmuir isotherms were constructed with the data from results of Figs 1(a–c), 2(a–c) and 3(d–f). In (**a–c**), A on the Y axis represents the biosorption amount (mg) when biosorption reached equilibrium. C on the X axis indicates the final metal concentration in the solution when biosorption reached equilibration. To determine the metal sorption and removal from the multi-component metal solution, and to detect energy spectra of the beads, the beads were pre-cultured for 48 h by shaking at 200 rpm in LPD and then fully rinsed by sterilized water before sorption. The sorption was conducted in the solution of unadjusted natural pH, and containing 5 g beads of a 3.2 mm diameter and 70 mg/L for each Cu, Pb and Cd by shaking incubation for 3 h at 200 rpm at 32 °C. Each datum in the Figure was the average mean ± SD from three independent experiments. The energy spectrum from the beads was recorded by the EDXS. EDXS, energy dispersive X-ray spectroscopy. LPD, liquid potato dextrose. PVA, polyvinyl alcohol. SA, sodium alginate. SD, standard deviation.

The Langmuir sorption isotherm model is to characterize the sorption kinetics^24^. Based on the data resulting from metal biosorption in the single metal solution (Fig. 2a–c), the change of biosorption of each metal by PVA-SA-conidia beads with initial metal concentration was described according to the Langmuir isotherm. As a result, biosorption for both Cu (Fig. 6a) and Cd (Fig. 6c) fit well with the Langmuir sorption isotherm model. However, the biosorption for Pb did not follow the typical Langmuir sorption model (Fig. 6b).

### Metal sorption capacity and removal efficiency in the multi-component metal solution

By using the PVA-SA-conidia beads in the multi-component metal solution of Cu, Pb, and Cd, the biosorption was in order of Pb > Cu > Cd. However, with the PVA-SA beads, the adsorption was in order of Cu > Pb > Cd (Fig. 6d). The removal rate by the PVA-SA-conidia beads was 87.7% for Pb, 85.8% for Cu, and 48% for Cd (Fig. 6d). The energy dispersive X-ray spectroscopy (EDXS) confirmed the preferential order and capacity of biosorption and adsorption in the multi-component metal solution, respectively, by the PVA-SA (Fig. 6e) and PVA-SA-conidia beads (Fig. 6f).

**Figure 7:**
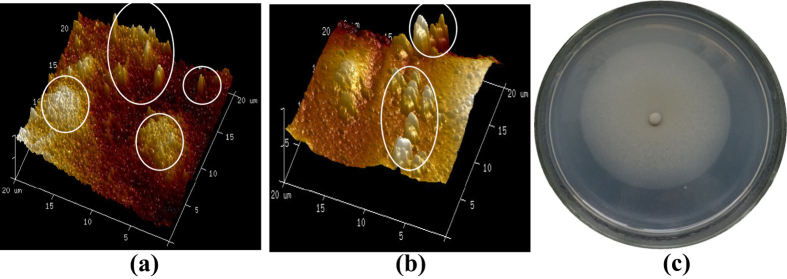
Outward growth of (**a,b**) and colony (**c**) formed by alive mycelium inside the PVA-SA-conidia beads after four cycles of successive sorption-desorption in the multi-component metal solution. (**a**) Surface of the beads from the parallel control treatment in the water without added metals, and (**b**) Surface of the beads after the fourth sorption in the solution containing 70 mg/L for each of Cu, Pb and Cd, Pb, where the cycled columnar projectures were the mycelia growing outward from the inside of the beads and imaged by AFM. The successive sorption-desorption was conducted following the procedures and conditions indicated in Fig. 4 except with no pre-culture of the beads before the first sorption. For the observation of formation of the colony, the beads after the fourth sorption were cultured for 7 d at 32 °C on PDA plates without added metals. The scale of the pictures in (**a,b**) was shown with a unit of μM. AFM, atomic force microscopy. PDA, potato dextrose agar. PVA, polyvinyl alcohol. SA, sodium alginate.

Additionally, the mycelia generated from PVA-SA-embedded GXCR conidia were still alive even after four cycles of successive sorption/desorption of the beads (Fig. 7).

## Discussion

The understanding of the mechanism in each biosorption system is a prerequisite for the engineering process application potential^25^. For immobilization of filamentous fungal biomass, a key step is to determine optimized immobilization conditions, depending on biomass types. To remove the heavy metals from aqueous solutions, most of the previous studies employed filamentous (mold-like) forms of the fungal biomass immobilized in forms of beads^26^,^27^,^28^,^29^,^30^, pieces^31^, columns^26^,^32^, discs^33^, or reticulated polyester foam biological support particles^32^, and these studies focused on equilibrium and kinetic studies. However, no attention was paid to changes in functional groups for metal biosorption by using filamentous fungi before and after biosorption, which represents an important chance to discover new groups capable of binding heavy metals. To our knowledge, there were only two reports, which employed the immobilized spores of filamentous fungus *Rhizopus* sp.^30^,^32^ to remove heavy metal from aqueous solutions.

In this study, we determined the optimal conditions for preparation of the beads to be 2% PVA, 3% SA, 1% H_3_BO_3_, 3% CaCl_2_, and 1.9 × 10^4^ conidia/mL for a cross-linking of 20 min to generate the beads to embed the conidia of heavy metal-resistant *P. janthinillum* strain GXCR. The further confirmed optimal conditions for high sorption and high removal efficiency by the PVA-SA-conidia beads included the solution of initial pH 3, pre-culture duration (72 h for both Cu and Pb biosorption; and 48 h for Cd biosorption) of the beads before sorption, sorption of 100 min, and bead size of 2.7 mm in diameter.

Under these conditions, the resulting beads were characterized by an ideal mechanical appropriate strength, a better settleability (Table 1), a better porosity formed by the mycelia grown from the immobilized conidia (Fig. 4d–h), and easy separation and recovery from the solution in particular for regeneration and reuse. These properties of the beads are really needed by metal removal processes from the solution in practice^23^. However, it is very difficult to make comparisons of our results with previous researches^3^,^13^,^26^,^27^,^28^,^29^,^30^,^31^,^32^,^33^ due to substantial differences in biosorption systems. Anyway, the biosorption system based on PVA-SA-conidia beads showed the higher metal biosorption especially in the multi-component metal solution, with the removal rate of 85.8% for Cu, 87.7% for Pb, and 48% for Cd (Fig. 6d).

The metal-resistant strains seem, no doubt, to be more suitable for application in the toxic heavy metal solution systems especially when the living cells are used^13^. The GXCR strain used in this study has a high resistance to multiple heavy metals^22^. This character made GXCR to grow very well in the biosorption systems (Fig. 4d–h).

Usually, metal biosorption by fungal biomass decreased as the temperature increased especially under high temperature^34^ because the high temperature can not only lead to distortion and/or damage of some functional sites on the cell surfaces and in the biomass^34^, but also affect the integrity of cell membranes, the stability of the metal-microorganism complex, the wall configuration of the microorganism cell, and the ionization of chemical moieties on the cell wall^23^. An unexpected finding was that changes in temperature form 15 to 40 °C did not substantially affect the biosorption and removal of Cu (Fig. 3d) and Pb (Fig. 3e) except Cd (Fig. 3f) by the PVA-SA-conidia beads, suggesting that embedding immobilization can prevent the above-mentioned high temperature-induced injury towards the living fungal cells.

Reportedly, higher fungal biomass concentrations in aqueous solutions lead to decrease in metal biosorption because of increase in the electrostatic interactions of the functional groups on the cell surfaces while accompanied by decreased cell surface area caused by attachment to each other^3^. In this study, when larger beads of over 2.7 mm in diameter were used the metal removal and biosorption were significantly decreased (Fig. 2d–f). One likely reason for this was associated with the increase in interference among the beads, on the other hand, surface-area to volume ratios were maybe reduced for larger beads.

Understanding of metal binding groups is an important foundation of a cell surface engineering approaches with the goal of enhancing biosorption efficiencies of heavy metal ions of microbial cells^35^. The live fungal biomass shows, to some extent, a highly selective adsorption^4^, with preferential biosorption order in multi-component metal solutions^4^,^32^,^36^. The preferential biosorption is associated with functional groups for metal biosorption. For example, carboxylate and amine were major functional groups for metal biosorption on *Aspergillus niger* biomass, but phosphate groups and the lipids fractions of the biomass do not participate in metal adsorption^37^. Cu seems to prefer amine sites in comparison with carboxylates^38^. The hydroxyl, amide, and acetyl groups are responsible for Cd biosorption by *Saccharomyces cerevisiae*^38^. In this study, functional groups newly found in biosorption by using the PVA-SA-conidia beads were CH_3_, PO_3_, and 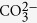 for both Cu and Pb biosorption, and 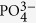 -PO_4_ for Cd biosorption (Table 2) when compared to Cu biosorption by immobilized cells of *Pycnoporus sanguineus* from aqueous solution^39^. Such discrepancies in recorded functional groups is likely associated with the differences fungal species, as well as biomass forms used (i.e. living vs. non-living), support materials for biomass immobilization, and possible surface modification of the biomass. Note that there were obvious differences of the functional groups in pattern of stretching vibration for metal sorption (Table 2), suggesting an importance of configuration of functional groups in metal sorption.

The metal bioaccumulation by GXCR mycelia have well been documented in our previous study^40^. Taken together with the alive mycelia generated from PVA-SA-conidia beads even after four cycles of successive sorption/desorption of the beads (Fig. 7), the metal sorption mechanisms by PVA-SA-conidia beads should include both biosorption and bioaccumulation by the mycelia.

With increasing temperature, the Cd biosorption decreased but removal rate significantly increased by using PVA-SA-conidia beads, (Fig. 3f). However, by using control PVA-SA beads, the Cd adsorption decreased, but the removal rate was not changed (Fig. 3f). These results together strongly suggest that there is active Cd sorption and desorption occurring on or in the control PVA-SA beads because of no relatively more functional groups capable of biding metals for PVA-SA matrix support.

After sorption/desorption, the very coarse surfaces of the mycelia inside of the PVA-SA-conidia beads (Fig. 4h) at least suggest effects of 0.5 M HCl used in desorption on the mycelia.

The Langmuir model is based on the assumption that maximum adsorption occurs when a saturated monolayer of solute molecules is present on the adsorbent surface^3^. The biosorption of Cu (Fig. 6a) and Cd (Fig. 6c) by PVA-SA-conidia beads fitted well the Langmuir model, suggesting that the biosorption should be mainly based on a single layer metal sorption as a molecular surface coverage, similar to that for an ion-exchange resin^41^. However, Pb biosorption by the PVA-SA-conidia beads did not follow the Langmuir model (Fig. 6b), suggesting that Pb biosorption is a multivariate process at least including surface- and metabolite-dependent sorption.

In conclusion, the PVA-SA-conidia beads showed high metal biosorption and removal from the aqueous solutions while also overcoming disadvantages during use the fugal biomass alone.

## Methods

### Fungal strains, media, and growth conditions

The filamentous fungus used was *P. janthinillum* strain GXCR^22^. The following media were used for cultivation of the fungus: conventional potato dextrose agar (PDA) and liquid potato dextrose (LPD) without agar. The conditions for cultivation of the fungus were dependent on experimental designs as indicated in the text.

### Immobilization of fungal conidia

Conidia produced by the fungal mycelia grown for 10 d at 32 °C on PDA plate were collected using 0.05% tween-80 and then suspended in sterilized water to make a conidial suspension of 4.0 × 10^7^ conidia/mL. Subsequently, the conidia were embedded following the methods in the literature^30^ but with some modifications. In brief, PVA and SA were completely dissolved by stirring in sterilized water in a 75 °C water bath, placed for 12 h at room temperature followed by an additional 1 h in a 75 °C water bath. Such operation procedures were repeated three times. After the last round of water bath treatment, the mix was allowed to cool to about 40 °C and then added by the prepared conidial suspension, resulting in the mix of 1.9 × 10^4^ conidia/mL. The resulting conidia-containing mix was gently injected into cross-linking solution throgugh a syringe of a No. 12 needle to produce the embedding beads. The cross-linking solution was composed of boric acid and calcium chloride. The control beads were in parallel prepared without the conidia. The beads were fully rinsed with sterilized water and then stored at 4 °C for use.

### Assay of the mechanical strength of the beads

Briefly, the air was slowly injected into the beads through a syringe with a No. 8 needle to apply pressure to the beads to observe the rate of their rupture. In addition, 10 randomly selected beads were placed on the microscope slide, a Petri dish were placed on the beads and then gradually added by analytical weights, and the total weight of the added weights were recorded when the beads deformed under loading weight. The mechanical strength of the beads was estimated and reported as average load weight per bead (g of a bead).

### Assay of metal sorption by the beads

The beads were pre-cultured for 1–4 d at 32 °C by shaking at 200 rpm in LPD medium, and then fully rinsed by sterilized water. Subsequently, the residual water present on surfaces of the beads was removed by using Whatman filter paper before biosorption. The fresh beads were put in the 250-mL flask containing 50 mL water solution with or without Cu, Pb, and Cd, and incubated at 200 rpm on a shaker. The metals in the solution were measured through the ZEEnit 700P atomic absorption spectrometry equipped with a flame graphite furnace (Analytik Jena, Germany). All sportion experiments were independently batch-repeated three times. The average values were used in this study.

### Regeneration and resue of the beads

After metal sorption, the beads were collected by filtration, soaked for 12 h in the distilled water and then desorbed for 24 h in 0.5 M HCl for regeneration of the beads. The regenerated beads were stored at 4 °C for reuse.

### Observation of the beads and analysis of energy spectrum

The beads were observed and photographed with an ordinary optical microscope. For observation of mycelial development inside the beads and analysis of the energy spectrum, the beads were cut with a scalpel, fixed for 24 h in 2.5% glutaraldehyde fixative, washed three times as 10 min for each time with 0.1 M phosphate buffer, subsequently dehydrated in gradient concentrations of ethanol, and then photographed to document fungal biomass morphology, while recording the energy spectra by using a HITACHI S-3400 scanning electron microscope (SEM) (Japan) equipped with an EDXS following the conventional procedures.

For the surfaces of the beads, the beads after sorption were rinsed three times with physiological saline, and then observed and photographed by a Dimension FastScan^TM^ atomic force microscopy (AFM) (Bruker, USA) under the three dimension (X-, Y- and Z-axles)-controlled parameters of 90 μm × 90 μm × 10 μm for II scanner, 35 μm × 35 μm × 3 μm for FastScan scanner, and the scanning speeds of >2 mm/s for Y-axle and 12 mm/s for Z-axle, respectively.

### Analysis of IS of the beads

The IS analysis followed the KBr method in the literature^42^ with minor modifications. In brief, the sample beads were pulverized using an agate mortar. The resulting sample powder was sieved through the 200-mesh plastic sieve. Two milligrams of the sieved sample were mixed with 200 mg of KBr powder, and then dried at 50 °C in an infrared oven. Subsequently, IS values of the samples were recorded in the range 4000–400 cm^−1^ on a Nicolet 6700 Fourier transform infrared (FT-IR) spectrophotometer.

### Statistical analysis

Statistical analyses were performed with SPSS. Differences between two variables in the mean were evaluated by the Student’s *t*-test at *p* value of <0.05.

## Additional Information

**How to cite this article**: Cai, C.-X. *et al.* A novel approach of utilization of the fungal conidia biomass to remove heavy metals from the aqueous solution through immobilization. *Sci. Rep.* **6**, 36546; doi: 10.1038/srep36546 (2016).

**Publisher’s note:** Springer Nature remains neutral with regard to jurisdictional claims in published maps and institutional affiliations.

## Figures and Tables

**Table 1:** The Orthogonal experiment design of production of the beads.

Experimental design	Experimental factor setting				Breakage rate of beads (%)	Description of beads
	PVA (%)	SA (%)	H_3_BO_3_ (%)	CaCl_2_ (%)		
1	2	2	2	2	Not counted	Unable to form beads
2	2	4	3	1	5	Easy to form beads
3	1	2	3	4	75	Easy to form beads
4	3	3	3	3	Not counted	Unable to form beads
5	1	1	1	1	Not counted	Unable to form beads
6	1	4	2	3	30	Difficult to form beads
7	3	1	2	4	Not counted	Unable to form beads
8	1	3	4	2	50	Easy to form beads
9	4	3	2	1	Not counted	Unable to form beads
10	2	3	1	4	0	Easy to form round beads; bad settleability
11	3	4	1	2	0	Easy to form round beads; better settleability
12	2	3	1	3	0	Easy to form round beads; better settleability
13	2	1	4	3	Not counted	Unable to form beads
14	4	1	3	2	Not counted	Unable to form beads
15	3	2	4	1	Not counted	Unable to form beads
16	4	4	4	4	Not counted	Unable to form beads
17	4	2	1	3	Not counted	Unable to form beads

H_3_BO_3_ and CaCl_2_ solutions were used as cross-linking solutions to prepare the beads. The cross-linking was conducted for 20 min, and cross-linking solution was renewed once during cross-linking. The breakage rate of the beads was counted from 50 beads after pressuring the beads through injecting air with a syringe with a No. 8 needle.

**Table 2:** Changes of IR peaks before and after metal biosorption, and functional groups for metal adsorption.

	Treatment		Structure adscription and stretching pattern of stretching vibration peaks
Wave number (cm^−1^)	**PVA-SA-conidia beads before Cu biosorption**	**PVA-SA-conidia beads after Cu biosorption**	
	3392.56	3420.39	-OH
	2955.39	No peak	CH_3_; Antisymmetric stretching vibration
	2927.16	2927.32	CH_2_; Antisymmetric stretching vibration
	No peak	1733.42	C = O
	1318.63	No peak	CH_2_; Outside rocking vibration
	No peak	1259.49	PO_3_; Antisymmetric stretching vibration
	890.84	882.09	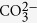 ; Outside bending vibration
	**PVA-SA-conidia before Pb biosorption**	**PVA-SA-conidia after Pb biosorption**	
Wave number (cm^−1^)	3392.56	3420.62	-OH
	2955.39	No peak	CH_3_; Antisymmetric stretching vibration
	2927.16	2927.87	CH_2_; Antisymmetric stretching vibration
	No peak	1733.46	C = O
	1318.63	1318.22	CH_2_; Outside rocking vibration
	No peak	1258.81	PO_3_; Antisymmetric stretching vibration
	890.84	882.21	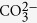 ; Outside bending vibration
	**PVA-SA-conidia before Cd biosorption**	**PVA-SA-conidia after Cd biosorption**	
Wave number (cm^−1^)	3392.56	3420.62	-OH
	2955.39	No peak	CH_3_; Antisymmetric stretching vibration
	2927.16	2926.50	CH_2;_ Antisymmetric stretching vibration
	No peak	1733.07	C = O
	1318.63	No peak	CH_2_; Outside rocking vibration
	1289.89	No peak	PO_3_; Antisymmetric stretching vibration
	No peak	1254.41	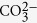 ; Outside bending vibration
	948.45	No peak	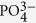 -PO_4_; Symmetric stretching vibration
